# Tobramycin-Linked Efflux Pump Inhibitor Conjugates Synergize Fluoroquinolones, Rifampicin and Fosfomycin against Multidrug-Resistant *Pseudomonas aeruginosa*

**DOI:** 10.3390/jcm7070158

**Published:** 2018-06-22

**Authors:** Xuan Yang, Ronald Domalaon, Yinfeng Lyu, George G. Zhanel, Frank Schweizer

**Affiliations:** 1 Department of Chemistry, University of Manitoba, Winnipeg, MB R3T 2N2, Canada; yangx315@myumanitoba.ca (X.Y.); umdomala@myumanitoba.ca (R.D.); lvyinfeng2010@163.com (Y.L.); 2 Institute of Animal Nutrition, Northeast Agricultural University, Harbin 150030, China; 3 Department of Medical Microbiology and Infectious Diseases, University of Manitoba, Winnipeg, MB R3A 1R9, Canada; ggzhanel@pcs.mb.ca

**Keywords:** tobramycin, efflux pump inhibitor, synergize, fluoroquinolones, rifampicin, fosfomycin, multidrug-resistant *Pseudomonas aeruginosa*

## Abstract

In this study, we examined the in vitro effect of tobramycin-efflux pump inhibitor (TOB-EPI) conjugates in combinations with fluoroquinolones, rifampicin and fosfomycin on the growth of multi-drug resistant (MDR) and extremely-drug resistant (XDR) *Pseudomonas aeruginosa*. The TOB-EPI conjugates include tobramycin covalently linked to 1-(1-naphthylmethyl)-piperazine (NMP) (**1**), paroxetine (PAR) (**2**) and a dibasic peptide analogue of MC-04,124 (DBP) (**3**). Potent synergism was found for combinations of TOB-NMP (**1**), TOB-PAR (**2**) or TOB-DBP (**3**) with either fluoroquinolones (moxifloxacin, ciprofloxacin), rifampicin or fosfomycin against a panel of multidrug-resistant/extensively drug-resistant (MDR/XDR) *P. aeruginosa* clinical isolates. In the presence of ≤8 mg/L (6.1–7.2 µM) (≤¼ × MIC_adjuvant_) concentration of the three conjugates, the MIC_80_ of moxifloxacin, ciprofloxacin, rifampicin and fosfomycin were dramatically reduced. Furthermore, the MIC_80_ of rifampicin (0.25–0.5 mg/L) and fosfomycin (8–16 mg/L) were reduced below their interpretative susceptibility breakpoints. Our data confirm the ability of TOB-NMP (**1**), TOB-PAR (**2**) and TOB-DBP (**3**) conjugates to strongly synergize with moxifloxacin, ciprofloxacin, rifampicin and fosfomycin against MDR/XDR *P. aeruginosa*. These synergistic combinations warrant further studies as there is an urgent need to develop new strategies to treat drug-resistant *P. aeruginosa* infections.

## 1. Introduction

**Figure 1:**
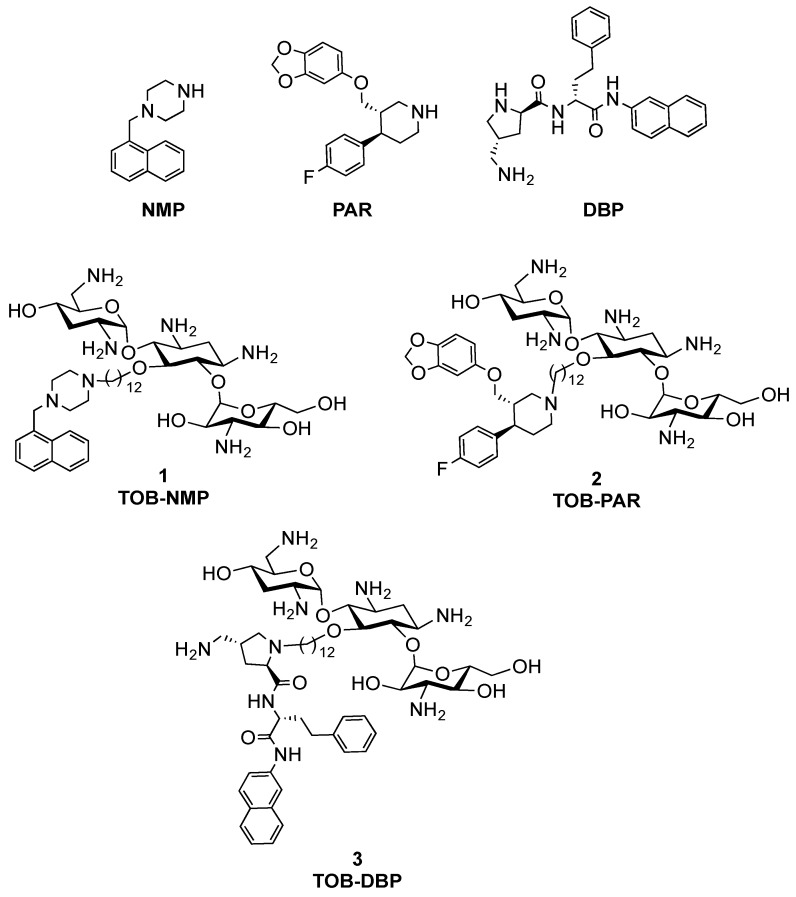
Structures of the efflux pump inhibitors (EPIs) 1-(1-naphthylmethyl)-piperazine (NMP), paroxetine (PAR), and a dibasic peptide analog of MC-04,124 (DBP) along with tobramycin-linked EPI conjugates **1**, **2** and **3**. TOB: tobramycin.

The opportunistic *Pseudomonas aeruginosa* is the leading cause of nosocomial and chronic lung infections in immunocompromised (e.g., cystic fibrosis) patients [1,2]. The World Health Organization (WHO) has listed carbapenem-resistant *P. aeruginosa* as one of the most critical (priority 1) pathogens that pose a serious threat to human health [3]. Among Gram-negative pathogens, infections caused by *P. aeruginosa* are particularly difficult to treat as the organism is both intrinsically resistant and capable of acquiring resistance (through mobile genetic elements) to most antibiotics [4]. The intrinsic resistance of *P. aeruginosa* is mostly due to its low outer membrane permeability, which is 12–100 times lower than that of *Escherichia coli*, presumably as a result of their relatively selective porins [4]. Overexpressed multidrug efflux pumps that limit the intracellular concentration of antibiotics is another key contributor of intrinsic resistance. Several small molecules such as 1-(1-naphthylmethyl)-piperazine (NMP) [5], paroxetine (PAR) [6,7] and DBP [8], the analogue of dibasic dipeptide d-Ala-d-hPhe-aminoquinoline (MC-04,124) (Figure 1), have been reported to inhibit efflux pumps in Gram-negative and/or Gram-positive bacteria, thereby restoring activity to legacy antibiotics. 

In a previous study, we discovered that linking a tobramycin (TOB) vector to the efflux pump inhibitors (EPIs) NMP, PAR, and DBP generated TOB-EPI conjugates (Figure 1) capable of sensitizing multidrug-resistant/extensively drug-resistant (MDR/XDR) Gram-negative bacilli, especially *P. aeruginosa*, to tetracycline antibiotics [9]. Mechanistic studies revealed tobramycin with a twelve carbon aliphatic chain (C_12_) to be a core fragment needed for outer membrane perturbation that leads to a ‘self-promoted’ uptake mechanism [9,10,11]. We also found that TOB-EPI conjugates are able to depolarize the inner membrane of *P. aeruginosa*, disrupting the electrical component (Δ*Ψ*) of bacterial proton motive force (PMF) that results in a compromised transmembrane chemical component (ΔpH) [9]. An increase in ΔpH would consequently facilitate the increased uptake of tetracyclines as the process of accumulation of tetracyclines is ΔpH-dependent [12]. Moreover, a compromised PMF affects PMF-dependent efflux systems that effectively negate the active efflux of susceptible antibiotics [9,10]. Herein, we describe the synergistic interactions of TOB-NMP (**1**), TOB-PAR (**2**) and TOB-DBP (**3**) with either fluoroquinolones (moxifloxacin and ciprofloxacin), rifampicin or fosfomycin against MDR/XDR *P. aeruginosa* clinical isolates.

## 2. Materials and Methods

### 2.1. Bacterial Strains

Clinically-relevant bacterial strains were collected from the Canadian National Intensive Care Unit (CAN-ICU) study [13] and Canadian Ward Surveillance (CANWARD) studies [14,15]. All isolates were transported to the reference laboratory (Health Sciences Centre, Winnipeg, MB, Canada) on Amies charcoal swabs, subcultured onto LB broth, and stocked in skim milk with 10% glycerol at −80 °C until antimicrobial susceptibility testing was carried out. The efflux pump deficient strains, *P. aeruginosa* PAO200 and *P. aeruginosa* PAO750, were provided by Dr. Ayush Kumar from University of Manitoba, Canada. All pathogens obtained from CAN-ICU and CANWARD studies have received ethics approval from the University of Manitoba Ethics Committee. In addition, participating Canadian health centers have obtained appropriate ethics approval to submit clinical specimens.

### 2.2. Antimicrobial Agents

Tobramycin sulfate, moxifloxacin hydrochloride, rifampicin, and ciprofloxacin hydrochloride were obtained from AK Scientific, Inc. (Union City, CA, USA). Fosfomycin sodium was obtained from Sigma-Aldrich (St. Louis, MO, USA). Glucose-6-phosphate (Sigma-Aldrich) was added to the medium at a final concentration of 25 mg/L for all evaluations of fosfomycin.

### 2.3. Antimicrobial Susceptibility Testing

The antimicrobial activity of the compounds against a panel of bacteria was evaluated by broth microdilution assay in accordance with the Clinical and Laboratory Standards Institute (CLSI) guidelines [16]. The overnight bacterial culture was diluted in saline to 0.5 McFarland turbidity, and then 1:50 diluted in Mueller−Hinton broth (MHB) for inoculation. The minimum inhibitory concentrations (MICs) of the antimicrobial agents were determined using 96-well plates containing doubling antimicrobial dilutions with MHB and incubated with equal volumes of inoculum for 18 h at 37 °C. The lowest concentration that inhibited visible bacterial growth was taken as the MIC for each antimicrobial agent which was also confirmed using EMax Plus microplate reader (Molecular Devices, San Jose, CA, USA) at a wavelength of 590 nm. We used a stock concentration of 10.24 mg/mL in deionized water or DMSO depending on the solubility of the compounds.

### 2.4. Antimicrobial Combination Screening

The checkerboard method [17] was used to assess synergism in all tested combinations. The fractional inhibitory concentration index (FICI) of each combination was calculated as follows: FICI is the sum of the fractional inhibitory concentration of antibiotic (FIC_antibiotic_) and fractional inhibitory concentration of adjuvant (FIC_ADJ_); FIC_antibiotic_ = MIC_combo_/MIC_antibiotic alone_; FIC_adjuvant_ = MIC_combo_/MIC_adjuvant alone_, where MIC_combo_ is the lowest inhibitory concentration of drug in the presence of the adjuvant; the combination is considered synergistic when the FICI is ≤0.5, no interaction is considered when the FICI is 0.5 FICI ≤ 4.0, and the combination is considered antagonistic when the FICI is 4.0 [18].

## 3. Results

We recently reported the preparation and biological evaluation of three TOB-EPI conjugates (Figure 1), namely TOB-NMP (**1**), TOB-PAR (**2**) and TOB-DBP (**3**) [9]. We found that the three conjugates were mostly inactive (MIC = 2–1024 mg/L) alone but significantly potentiated minocycline, in combination, against MDR/XDR *P. aeruginosa* clinical isolates [9]. Preliminary results indicated that the adjuvant properties of **1**–**3** against *P. aeruginosa* are not limited to tetracycline antibiotics and can also be extended to other antimicrobial classes [9]. Herein, we further expand our understanding on the adjuvant properties of the three TOB-EPI conjugates to other antibacterial classes including rifampicin, fluoroquinolones (ciprofloxacin and moxifloxacin) and fosfomycin.

Aligned with our previous results with minocycline [9], the *P. aeruginosa* inactive efflux pump inhibitors NMP and PAR displayed no interaction (FICI = 0.63, 1.02) with rifampicin (Table 1). On the other hand, the *P. aeruginosa* active efflux pump inhibitor DBP was synergistic (FICI = 0.09) with rifampicin (Table 1) against wild-type *P. aeruginosa* PAO1. The absolute MIC of rifampicin (MIC = 32 mg/L) in combination with 8 mg/L (6.1–7.2 µM) of either **1**, **2**, **3** or DBP was found to be ≤0.25, ≤0.25, ≤0.25 and 4 mg/L, respectively. Indeed, a ≥128-fold potentiation of rifampicin was observed for the three conjugates relative to a meager 8-fold potentiation induced by DBP. However, we did not observe synergy of rifampicin with tobramycin (FICI = 1.0) in wild-type *P. aeruginosa* PAO1.

Since fluoroquinolones are good substrates for *P. aeruginosa* RND efflux pumps [19,20], we expanded our studies to combinations of TOB-EPI conjugates (**1**, **2** or **3**) or efflux pump inhibitors (NMP, PAR or DBP) with the fluoroquinolone antibiotic moxifloxacin against wild-type *P. aeruginosa* PAO1 (Table 1). Moxifloxacin was strongly potentiated by tobramycin-linked EPI conjugates **1** (FICI = 0.16), **2** (FICI = 0.19) and **3** (FICI = 0.31). However, as a control, the combination study of moxifloxacin with tobramycin against *P. aeruginosa* PAO1 strain was not synergistic (FICI = 1.1). No synergistic effect was observed for NMP (FICI = 2.00) nor PAR (FICI = 2.00), whereas synergy was found for DBP (FICI = 0.19). The absolute MICs of moxifloxacin (MIC = 2 mg/L) in the presence of 8 mg/L conjugates **1**, **2** or **3** were found to be 0.125, 0.063 and 0.063 mg/L, respectively (Table 1). Thus, 8–16 fold potentiation of moxifloxacin was observed for the three conjugates. All three TOB-EPI conjugates (**1**, **2** or **3**) and DBP also displayed strong synergism with fosfomycin (FIC index of 0.09–0.25) (Table 1). At 8 mg/L of TOB-EPI conjugates (**1**, **2** or **3**), the absolute MICs of fosfomycin (MIC = 32 mg/L) were reduced to 1, 2 and 1 mg/L, respectively (Table 1). Thus, 8–32 fold potentiation of fosfomycin was observed for the three conjugates. As a control, a combination study of fosfomycin with tobramycin was performed and the result indicated no synergistic effect against wild-type *P. aeruginosa* PAO1 strain (FICI = 1.0).

Prompted by our findings in wild-type *P. aeruginosa* strain, we further assessed the synergism of the three TOB-EPI conjugates **1**, **2** and **3** in combination with either moxifloxacin, ciprofloxacin, rifampicin or fosfomycin against a panel of eight MDR/XDR *P. aeruginosa* clinical isolates. These *P. aeruginosa* isolates are resistant to many antibiotics as shown in Supplementary Data Table S1, to which all but one are ciprofloxacin-resistant. All the three conjugates were found to be synergistic with the four tested antibiotics (Table 2). Both TOB-NMP (**1**) and TOB-PAR (**2**) strongly potentiated moxifloxacin (4–128 fold), ciprofloxacin (4–256 fold), rifampicin (32–128 fold), and fosfomycin (2–64 fold) against all tested MDR/XDR *P. aeruginosa* strains. Similar results were observed for the combinations of TOB-DBP (**3**) and moxifloxacin. TOB-DBP (**3**) also potentiated ciprofloxacin, rifampicin and fosfomycin against most of the strains tested. However, TOB-DBP (**3**) displayed no interactions with ciprofloxacin (FICI = 0.75) and rifampicin (FICI = 0.516) against *P. aeruginosa* PA260-97103 strain. Fosfomycin in combination with TOB-DBP (**3**) displayed no interaction (FICI = 0.75) against *P. aeruginosa* PA262-101856 strain.

We further assessed the potency of TOB-EPI conjugates as adjuvants by comparing the absolute MICs of the four antibiotics, in the presence of ≤8 mg/L (6.1–7.2 µM) (≤¼ × MIC_adjuvant_) conjugates, to established susceptibility breakpoints. According to the Clinical and Laboratory Standards Institute (CLSI) [21], the susceptibility breakpoint of ciprofloxacin for *Pseudomonas aeruginosa* is ≤1 mg/L. However, no established susceptibility breakpoint of moxifloxacin, rifampicin and fosfomycin exists for *Pseudomonas* spp., and therefore we used other breakpoints in other organisms for comparison. We interpreted susceptibility to moxifloxacin for *Pseudomonas aeruginosa* to be similar to the established one for ciprofloxacin, as both belong to the fluoroquinolone class of antibiotics. CLSI denotes susceptibility to rifampicin for *Enterococcus* spp. as ≤1 mg/L [21]. Conversely, susceptibility to fosfomycin was described to be ≤64 mg/L for Enterobacteriaceae [21].

**Figure 2:**
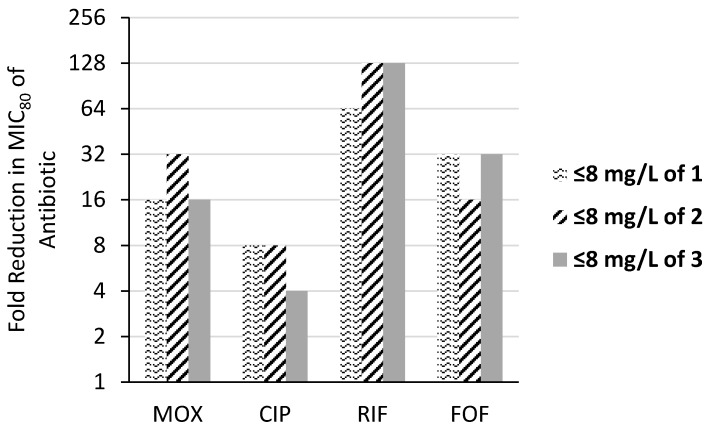
TOB-EPIs (**1**, **2** or **3**) potentiate the activity of moxifloxacin (MOX), ciprofloxacin (CIP), rifampicin (RIF) and fosfomycin (FOF) against a panel of MDR/XDR *P. aeruginosa* clinical isolates (*n* = 8). The MIC_80_ of MOX, CIP, RIF and FOF were significantly reduced in the presence of ≤8 mg/L (6.1–7.2 µM) (≤¼ × MIC_adjuvant_) of the corresponding potentiator (**1**, **2**, or **3**).

Next, we studied whether the absolute MIC of the four antibiotics in the presence of the three TOB-EPI conjugates at ≤8 mg/L (6.1–7.2 µM) (≤¼ × MIC_adjuvant_) reaches the expected susceptibility breakpoint of ciprofloxacin and moxifloxacin. Our results (Table 2) show that in 6/8 cases, the adjuvants cannot reach the expected susceptibility breakpoint of the two fluoroquinolone antibiotics. The two *P. aeruginosa* strains which reach the susceptibility breakpoint (91433 and 101243) do not contain DNA gyrase A mutation, indicating that fluoroquinolone resistance is mostly due to active efflux in these strains [11]. Out of the two fluoroquinolones, moxifloxacin seemed to be strongly potentiated by the conjugates relative to ciprofloxacin (Figure 2). In contrast, the MIC of rifampicin was reduced below the susceptibility breakpoint in all strains tested by conjugates **1** and **2** (Table 2). However, conjugate **3** was able to reduce the MIC of rifampicin below the susceptibility breakpoint for all strains except *P. aeruginosa* PA260-97103 (absolute MIC = 16 mg/L). All the three conjugates lowered the absolute MIC of fosfomycin in all strains tested except *P. aeruginosa* 100036.

The MIC_80_ of moxifloxacin, ciprofloxacin, rifampicin and fosfomycin in combination with ≤8 mg/L (6.1–7.2 µM) (≤¼ × MIC_adjuvant_) TOB-EPIs conjugates (**1**, **2**, or **3**) against the tested *P. aeruginosa* panel were significantly lower than the MIC_80_ of the antibiotic alone (Table 3 and Figure 2). More importantly, the absolute MIC_80_ of rifampicin and fosfomycin were below their respective susceptibility breakpoints. In the presence of ≤8 mg/L (7.2 µM) (≤¼ × MIC_adjuvant_) TOB-NMP (**1**), the absolute MIC_80_ of rifampicin was 0.5 mg/L while that of fosfomycin was 8 mg/L. The absolute MIC_80_ of rifampicin and fosfomycin in the presence of ≤8 mg/L (6.9 µM) (≤¼ × MIC_adjuvant_) TOB-PAR (**2**) was found to be 0.25 mg/L and 16 mg/L. Similarly, the absolute MIC_80_ of rifampicin and fosfomycin in the presence of ≤8 mg/L (6.1 µM) (≤¼ × MIC_adjuvant_) TOB-DBP (**3**) was 0.25 mg/L and 8 mg/L.

Considering the possible effect of tobramycin-efflux pump inhibitor conjugates on the active efflux of fluoroquinolones, we assessed the synergy of moxifloxacin and the three conjugates in efflux-deficient *P. aeruginosa* strains (Table 4). PAO200 is a MexAB−OprM deletion strain while PAO750 is an efflux-sensitive strain that lacks five different clinically relevant RND pumps (MexAB−OprM, MexCD−OprJ, MexEF−OprN, MexJK, and MexXY) and the OM protein OpmH [22]. These efflux pumps confer resistance on *P. aeruginosa* by expelling a wide variety of antibiotic substrates including quinolones, tetracyclines and others. As expected, a significant reduction in MIC of moxifloxacin was observed for PAO200 (MIC = 0.125 mg/L) and PAO750 (MIC = 0.008 mg/L) as active efflux contributes greatly to fluoroquinolone resistance. Interestingly, a 16-fold MIC reduction was observed for TOB-NMP (**1**) from wild-type *P. aeruginosa* PAO1 (MIC = 128 mg/L) to PAO750 (MIC = 8 mg/L) while only a 2- to 4-fold difference was observed for the MIC of TOB-PAR (**2**) and TOB-DBP (**3**) against PAO1, PAO200 and PAO750. The combination of conjugate **1** and moxifloxacin remained synergistic across the efflux-deficient strains, albeit weakly synergistic (FICI = 0.31) against *P. aeruginosa* PAO750. Both conjugates **2** (FICI = 0.19) and **3** (FICI = 0.25) were found to be synergistic with moxifloxacin against the MexAB-OprM-deficient PAO200 strain. However, no interaction was found between moxifloxacin and conjugates **2** (FICI = 0.63) or **3** (FICI = 0.63) against PAO750.

## 4. Discussion

The low permeability of the outer membrane and overexpressed multidrug efflux pumps in Gram-negative bacteria, especially in *P. aeruginosa*, limits effective antibiotics for treatment [23]. The compounding effect of the restrictive lipid bilayer and active efflux prevents the intracellular accumulation of antibiotics to concentrations needed to achieve biological effect. The problem is further exacerbated in drug-resistant organisms as they express genetically encoded resistance mechanism that may actively incapacitate antibiotics. Unfortunately, no new antibiotics with a novel mode of action for Gram-negative bacteria have been introduced in the clinic for more than five decades. There is a definite need to develop new strategies which are able to overcome resistance in Gram-negative pathogens, for which the combination therapy of existing antibiotics with adjuvants is a promising option [24].

We recently described the preparation of TOB-EPI conjugates (**1**, **2** or **3**) that synergize tetracycline antibiotics [9]. Moreover, we also demonstrated their ability to permeabilize the outer membrane of *P. aeruginosa* in a dose-dependent manner [9]. Herein, TOB-EPI conjugates (**1**, **2** or **3**) were found to significantly potentiate the outer membrane impermeable rifampicin (32–128 fold) against a panel of MDR/XDR *P. aeruginosa* clinical isolates. At ≤8 mg/L (6.1–7.2 µM) (≤¼ × MIC_adjuvant_) concentration of either the three conjugates, the absolute MIC_80_ of rifampicin was significantly reduced below susceptibility breakpoint. This suggest that conjugates **1**, **2** and **3** are good candidates for future adjuvant therapy development in combination with rifampicin. As rifampicin is a poor substrate for *P. aeruginosa* RND efflux pumps [9,10], membrane permeabilization may be responsible for the observed synergism with TOB-EPI conjugates. The *P. aeruginosa* inactive efflux pump inhibitors NMP and PAR were found to exhibit no interactions with rifampicin. In contrast, the *P. aeruginosa* active DBP was found to be synergistic with rifampicin against wild-type *P. aeruginosa* PAO1. A previous report of DBP analog PAβΝ revealed its ability to permeabilize bacterial membranes in a concentration-dependent manner [25], therefore this may have contributed to the observed rifampicin potentiation.

All three TOB-EPI conjugates strongly potentiated (fluoroquinolones (moxifloxacin 4–128 fold or ciprofloxacin 4–256 fold) against wild-type, fluoroquinolone-resistant and MDR/XDR *P. aeruginosa*. Out of the two fluoroquinolones tested, combinations of the three TOB-EPI conjugates with moxifloxacin yielded stronger potentiation relative to ciprofloxacin (Figure 2). However, the conjugates were not able to bring down the absolute MIC_80_ of both fluoroquinolones below their susceptibility breakpoint. It should be noted that the MICs of both fluoroquinolones were reduced below the susceptibility breakpoint in only two isolates (91433 and 101243 isolates which lack T^83^ to I^83^ mutation). This suggests that the conjugates enhance the intracellular concentration of fluoroquinolones. However, this effect cannot compensate acquired resistance caused by genetic mutations of the target enzyme.

The synergy of the conjugates with fluoroquinolones may not only be attributed to adjuvant-induced enhanced membrane permeability but may also be due to a compromised activity of PMF-dependent efflux pumps. We recently demonstrated that the TOB-EPI conjugates strongly reduce motility at sub-MIC concentration and disrupt the electrical component (Δ*Ψ*) of the PMF [9]. This action in turn may affect efflux systems that are dependent to PMF, leading to reduced efflux of fluoroquinolones. Our data revealed that the three conjugates were poor substrates of the MexAB-OprM RND efflux pump (Table 4). However, TOB-NMP (**1**) may be a substrate of other efflux systems in *P. aeruginosa* since a 16-fold MIC reduction was observed from wild-type PAO1 to the multiple efflux pump-deficient PAO750. We found that the synergism between moxifloxacin and TOB-EPI conjugates was independent of the MexAB-OprM RND efflux pump. Yet, there was a clear effect on the tested combinations of moxifloxacin and TOB-EPI conjugates against PAO750. The potent synergistic interaction with moxifloxacin found against wild-type PAO1 were drastically reduced to either weakly synergistic (for conjugate **1**) or no interaction (for conjugates **2** and **3**) against PAO750. Therefore, we assume that either MexCD-OprJ, MexEF-OprN, MexXY, or MexJK efflux pumps is affected by the TOB-EPI conjugates action on PMF. Certainly, moxifloxacin is a good substrate of many efflux pumps in *P. aeruginosa*.

Fosfomycin is a bactericidal antibiotic that inhibits cell wall biosynthesis [26]. Specifically, fosfomycin inactivates the enzyme UDP-*N*-acetylglucosamine enolpyruvyl transferase (MurA) that catalyzes the formation of peptidoglycan precursor UDP *N*-acetylmuramic acid (UDP-MurNAc) [26,27]. The three TOB-EPI conjugates strongly potentiated the activity of fosfomycin (2–64 fold) against wild-type and MDR/XDR *P. aeruginosa* clinical isolates susceptible or resistant to fosfomycin. In the presence of a ≤8 mg/L (6.1–7.2 µM) (≤¼ × MIC_adjuvant_) concentration of the conjugates, the absolute MIC for 7/8 isolates was ≤16 mg/L, which is 4-fold lower than the expected susceptibility breakpoint of fosfomycin (≤64 mg/L). Fosfomycin is known to be a poor substrate of the multidrug efflux system in *P. aeruginosa* [28] and it is understood that its cellular entry occurs through porins [29]. We hypothesize that the observed synergy of fosfomycin with TOB-EPI adjuvants reflects the enhanced cellular permeation of fosfomycin via the self-promoted uptake of TOB-EPI adjuvants.

## 5. Conclusions

In conclusion, we demonstrate promising synergistic combinations of TOB-EPI conjugates with either fluoroquinolones, rifampicin or fosfomycin against MDR/XDR *P. aeruginosa*. More importantly, the conjugates TOB-NMP (**1**), TOB-PAR (**2**) and TOB-DBP (**3**) significantly reduced the MIC_80_ of rifampicin and fosfomycin below their respective susceptibility breakpoints. These findings show that the adjuvant potency of TOB-EPI conjugates is not limited to tetracyclines [9] but can be expanded to other legacy antibiotics.

## Figures and Tables

**Table 1:** Combination studies of TOB-EPIs (**1**, **2**, or **3**) or EPIs (NMP, PAR or DBP) with moxifloxacin (MOX), rifampicin (RIF) or fosfomycin (FOF) against wild-type *P. aeruginosa* PAO1 strain.

Antibiotic	MIC_antibiotic alone_ (mg/L)	Adjuvant (ADJ)	MIC_ADJ alone_ (mg/L)	FICI	Absolute MIC *^a^* (mg/L)	Potentiation (fold) *^b^*
MOX	1	**1**	128	0.16	0.125	8
MOX	1	NMP	512	2.00	1	1
MOX	2	**2**	32	0.19	0.063	32
MOX	1	PAR	256	2.00	1	1
MOX	1	**3**	32	0.31	0.063	32
MOX	1	DBP	128	0.19	0.25	4
RIF	32	**1**	128	0.04	≤0.25	≥128
RIF	32	NMP	512	1.02	32	1
RIF	32	**2**	64	0.03	≤0.25	≥128
RIF	32	PAR	512	0.63	32	1
RIF	32	**3**	16	0.08	≤0.25	≥128
RIF	32	DBP	256	0.09	4	8
FOF	32	**1**	64	0.09	1	32
FOF	16	NMP	512	1.00	16	1
FOF	16	**2**	32	0.25	2	8
FOF	16	PAR	256	0.75	16	1
FOF	32	**3**	32	0.13	1	32
FOF	16	DBP	128	0.16	1	16

*^a^* Absolute MIC of antibiotic in the presence of 8 mg/L (6.1–7.2 µM) of corresponding potentiator. *^b^* Antibiotic activity potentiation at 8 mg/L (6.1–7.2 µM) of corresponding potentiator. FICI: fractional inhibitory concentration index.

**Table 2:** Combination studies of TOB-EPIs (**1**, **2**, or **3**) with moxifloxacin (MOX), ciprofloxacin (CIP), rifampicin (RIF) or fosfomycin (FOF) against MDR/XDR *P. aeruginosa* clinical isolates.

*P. aeruginosa*	Antibiotic	MIC_antibiotic alone_ (mg/L)	Adjuvant (ADJ)	MIC_ADJ alone_ (mg/L)	FICI	Absolute MIC (mg/L)
PA262-101856	MOX	64	**1**	64	0.188	8 *^a^*
PA262-101856	MOX	128	**2**	32	0.125	2 *^a^*
PA262-101856	MOX	128	**3**	32	0.188	4 *^a^*
PA262-101856	CIP	32	**1**	64	0.188	4 *^a^*
PA262-101856	CIP	32	**2**	32	0.250	4 *^a^*
PA262-101856	CIP	32	**3**	64	0.250	4 *^a^*
PA262-101856	RIF	1024	**1**	128	0.047	4 *^a^*
PA262-101856	RIF	1024	**2**	32	0.070	≤2 *^a^*
PA262-101856	RIF	1024	**3**	64	0.078	≤2 *^a^*
PA262-101856	FOF	8	**1**	128	0.141	1 *^a^*
PA262-101856	FOF	8	**2**	32	0.375	2 *^a^*
PA262-101856	FOF	8	**3**	64	0.750	8 *^a^*
PA260-97103	MOX	64	**1**	2	0.250	2 *^b^*
PA260-97103	MOX	128	**2**	8	0.188	2 *^b^*
PA260-97103	MOX	64	**3**	4	0.500	16 *^b^*
PA260-97103	CIP	32	**1**	2	0.500	8 *^b^*
PA260-97103	CIP	16	**2**	16	0.250	≤0.125 *^a^*
PA260-97103	CIP	32	**3**	4	0.750	16 *^b^*
PA260-97103	RIF	16	**1**	2	0.375	0.5 *^a^*
PA260-97103	RIF	16	**2**	16	0.070	≤0.125 *^a^*
PA260-97103	RIF	16	**3**	4	0.516	16 *^b^*
PA260-97103	FOF	8	**1**	4	0.188	≤0.031 *^b^*
PA260-97103	FOF	8	**2**	16	0.125	≤0.031 *^a^*
PA260-97103	FOF	4	**3**	4	0.375	0.5 *^b^*
100036	MOX	128	**1**	256	0.078	8 *^a^*
100036	MOX	128	**2**	64	0.094	4 *^a^*
100036	MOX	128	**3**	32	0.188	2 *^a^*
100036	CIP	64	**1**	256	0.156	8 *^a^*
100036	CIP	64	**2**	64	0.250	8 *^a^*
100036	CIP	64	**3**	32	0.313	8 *^a^*
100036	RIF	16	**1**	256	0.023	≤0.125 *^a^*
100036	RIF	16	**2**	128	0.047	≤0.125 *^a^*
100036	RIF	16	**3**	32	0.070	≤0.125 *^a^*
100036	FOF	1024	**1**	256	0.063 x 0.313	512 *^a^*
100036	FOF	1024	**2**	128	0.125 x 0.625	512 *^a^*
100036	FOF	1024	**3**	32	0.250 x 0.375	128 *^a^*
101885	MOX	64	**1**	256	0.141	8 *^a^*
101885	MOX	64	**2**	64	0.188	4 *^a^*
101885	MOX	64	**3**	8	0.375	8 *^b^*
101885	CIP	32	**1**	256	0.258	8 *^a^*
101885	CIP	32	**2**	32	0.375	4 *^a^*
101885	CIP	32	**3**	8	0.500	8 *^b^*
101885	RIF	16	**1**	256	0.031	≤0.125 *^a^*
101885	RIF	16	**2**	32	0.125	≤0.125 *^a^*
101885	RIF	16	**3**	16	0.094	≤0.125 *^a^*
101885	FOF	32	**1**	256	0.125	4 *^a^*
101885	FOF	32	**2**	32	0.188	4 *^a^*
101885	FOF	32	**3**	32	0.125	2 *^a^*
PA259-96918	MOX	512	**1**	1024	0.031 x 0.033	16 *^a^*
PA259-96918	MOX	1024	**2**	512	0.008 x 0.031	16 *^a^*
PA259-96918	MOX	512	**3**	64	0.047	4 *^a^*
PA259-96918	CIP	256	**1**	1024	0.063 x 0.066	16 *^a^*
PA259-96918	CIP	512	**2**	512	0.063 x 0.039	32 *^a^*
PA259-96918	CIP	256	**3**	64	0.125	16 *^a^*
PA259-96918	RIF	16	**1**	1024	0.008 x 0.009	≤0.125 *^a^*
PA259-96918	RIF	16	**2**	512	0.008 x 0.012	≤0.125 *^a^*
PA259-96918	RIF	16	**3**	32	0.039	≤0.125 *^a^*
PA259-96918	FOF	8	**1**	1024	0.063	0.5 *^a^*
PA259-96918	FOF	8	**2**	512	0.063 x 0.094	1 *^a^*
PA259-96918	FOF	16	**3**	64	0.094	0.5 *^a^*
PA264-104354	MOX	128	**1**	128	0.078	8 *^a^*
PA264-104354	MOX	128	**2**	64	0.156	2 *^a^*
PA264-104354	MOX	128	**3**	16	0.125	1 *^a^*
PA264-104354	CIP	32	**1**	128	0.250	8 *^a^*
PA264-104354	CIP	32	**2**	64	0.313	8 *^a^*
PA264-104354	CIP	32	**3**	16	0.250	2 *^a^*
PA264-104354	RIF	32	**1**	128	0.020	≤0.125 *^a^*
PA264-104354	RIF	32	**2**	64	0.063	≤0.125 *^a^*
PA264-104354	RIF	32	**3**	8	0.129	≤0.125 *^a^*
PA264-104354	FOF	8	**1**	128	0.125	0.5 *^a^*
PA264-104354	FOF	16	**2**	64	0.125	0.5 ^a^
PA264-104354	FOF	16	**3**	32	0.094	0.5 *^a^*
91433	MOX	8	**1**	32	0.156	0.25 *^a^*
91433	MOX	8	**2**	16	0.500	0.5 *^a^*
91433	MOX	8	**3**	8	0.281	0.25 *^b^*
91433	CIP	2	**1**	32	0.250	0.125 *^a^*
91433	CIP	2	**2**	16	0.500	0.125 *^a^*
91433	CIP	2	**3**	8	0.266	0.031 *^b^*
91433	RIF	16	**1**	16	0.375	0.25 *^a^*
91433	RIF	16	**2**	32	0.188	0.25 *^a^*
91433	RIF	16	**3**	8	0.250	≤0.125 *^b^*
91433	FOF	4	**1**	16	0.188	0.25 *^a^*
91433	FOF	2	**2**	32	0.375	≤0.25 *^a^*
91433	FOF	2	**3**	8	0.375	0.25 *^b^*
101243	MOX	4	**1**	64	0.125	0.25 *^a^*
101243	MOX	4	**2**	32	0.250	0.125 *^a^*
101243	MOX	4	**3**	16	0.156	≤0.0625 *^a^*
101243	CIP	2	**1**	64	0.281	0.5 *^a^*
101243	CIP	2	**2**	32	0.375	0.5 *^a^*
101243	CIP	2	**3**	16	0.188	0.125 *^a^*
101243	RIF	8	**1**	64	0.063	≤0.0625 *^a^*
101243	RIF	8	**2**	32	0.125	≤0.0625 *^a^*
101243	RIF	16	**3**	16	0.094	≤0.031 *^a^*
101243	FOF	256	**1**	64	0.125	8 *^a^*
101243	FOF	256	**2**	32	0.188	16 *^a^*
101243	FOF	256	**3**	32	0.047	4 *^a^*

*^a^* Absolute MIC of antibiotic in the presence of 8 mg/L (6.1–7.2 µM) of corresponding adjuvant. *^b^* Absolute MIC of antibiotic in the presence of ¼ × MIC of corresponding adjuvant.

**Table 3:** In vitro activity of moxifloxacin (MOX), ciprofloxacin (CIP), rifampicin (RIF) and fosfomycin (FOF) alone or in combination with fixed concentration (≤8 mg/L (6.1–7.2 µM)) of TOB-EPIs (**1**, **2**, or **3**) against MDR/XDR *P. aeruginosa* clinical isolates (*n* = 8).

Antimicrobial	MIC_50_ (mg/L)	MIC_80_ (mg/L)	Range (mg/L)
MOX	64	128	4–512
CIP	32	64	2–512
RIF	16	32	8–1024
FOF	16	256	2–256
**1**	64	256	2–1024
**2**	32	128	8–512
**3**	16	32	4–64
MOX + **1**	8	8	0.25–16
MOX + **2**	2	4	0.125–16
MOX + **3**	2	8	0.06–16
CIP + **1**	8	8	0.125–8
CIP + **2**	4	8	0.125–32
CIP + **3**	4	16	0.03–16
RIF + **1**	0.125	0.5	0.06–4
RIF + **2**	0.125	0.25	0.06–2
RIF + **3**	0.125	0.25	0.03–2
FOF + **1**	0.5	8	0.03–512
FOF + **2**	1	16	0.03–512
FOF + **3**	0.5	8	0.25–128

**Table 4:** In vitro activity of moxifloxacin (MOX), TOB-EPIs (**1**, **2** and **3**) and combinations of thereof against wild-type *P. aeruginosa* PAO1 and efflux pump deficient PAO200 and PAO750 strains.

Strain	MIC (mg/L)				FICI		
	MOX	1	2	3	MOX + 1	MOX + 2	MOX + 3
PAO1	1	128	32	32	0.16	0.19	0.31
PAO200	0.125	128	32	16	0.08	0.19	0.25
PAO750	0.008	8	8	8	0.31	0.63	0.63

PAO200 strain: PAO1, ΔmexAB-oprM; PAO750 strain: PAO1, ΔmexAB-oprM, ΔmexCD-oprJ, ΔmexEF-oprN, ΔmexXY, ΔmexJK, ΔopmH.

## Supplementary Materials

The following is available online at http://www.mdpi.com/2077-0383/7/7/158/s1, Table S1: antimicrobial susceptibility profile of *P. aeruginosa* clinical isolates used in this study.
