# Supplementary material for: Tobramycin-Linked Efflux Pump Inhibitor Conjugates Synergize Fluoroquinolones, Rifampicin and Fosfomycin against Multidrug-Resistant Pseudomonas aeruginosa
Source: J Clin Med. 2018 Jun 22;7(7):158. doi: 10.3390/jcm7070158 (PMC6069439; doi:10.3390/jcm7070158)
Supplement: Supplementary file 1 [file jcm-07-00158-s001.pdf]

Table S1. Antimicrobial susceptibility profile of *P. aeruginosa* clinical isolates used in this study

Stock #	PTZ	A/C	AZT	CFZ	CTR	CPM	CAZ	IMI	MER	DOR	ETP	TOB	GEN	AMK	TGC	DOX	CST	SXT
100036	8	>32	8	>128	32	4	8	8	4	4	>32	64	>32	32	>16	>32	2	>8
101885[1]	16	>32	32	>128	32	8	8	1	1	0.5	32	≤0.5	≤0.5	≤1	8	>32	1	>8
PA259-96918	64	>32	16	>128	>64	>64	>32	32	>32	>32	>32	>64	>32	>64	8	16	1	>8
PA260-97103	128	>32	16	>128	>64	16	32	32	16	16	>32	32	>32	4	16	16	1	2
PA262-101856	64	>32	32	>128	64	32	16	32	32	8	>32	>64	>32	>64	>16	>32	1	>8
PA264-104354	256	>32	64	>128	>64	32	>32	32	>32	16	>32	64	>32	8	>16	>32	1	>8
91433	64	>32	ND	>128	>64	16	>32	32	8	8	>32	8	32	>32	>16	32	4	1
101243	128	>32	ND	>128	>64	64	>32	16	16	16	>32	≥64	>32	>64	8	4	1024	4

PTZ: piperacillin-tazobactam; A/C: amoxicillin-clavulanic acid; AZT: aztreonam; CFZ: cefazolin; CTR: ceftriaxone; CPM: cefepime; CAZ: ceftazidime; IMI: imipenem; MER: meropenem; DOR: doripenem; ETP: ertapenem; TOB: tobramycin; GEN: gentamicin; AMK: amikacin; TGC: tigecycline; DOX: doxycycline; CST: colistin; SXT: trimethoprim-sulfamethoxazole; ND: not determined; [1] non MDR/XDR strain
